# Endocarditis Without Risk Factors: Clinical Awareness Is Key

**DOI:** 10.7759/cureus.46988

**Published:** 2023-10-13

**Authors:** Ines Pedrosa, Ana L Costa, Ana Reis e Melo, Margarida Tavares

**Affiliations:** 1 Department of Pediatrics, Centro Hospitalar de Leiria, Leiria, PRT; 2 Department of Pediatric Cardiology, Centro Hospitalar Universitário de São João, Porto, PRT; 3 Pediatric Infectious Diseases and Primary Immunodeficiencies Unit, Centro Hospitalar Universitário de São João, Porto, PRT

**Keywords:** valvular vegetation, cerebral septic emboli, septic emboli, infectious diseases, infective endocarditis

## Abstract

Infective endocarditis (IE) is a relatively common disease that can manifest as a spectrum of clinical findings. Clinical awareness is key for the diagnosis.

We present a case of a 14-year-old adolescent with fever, coughing, skin lesions, lip drooping, and quadrantanopia. Lumbar puncture was unremarkable and a head CT scan showed ischemic lesions. Blood cultures were positive for *Staphylococcus aureus*. A transesophageal echocardiogram showed a 7 x 7 mm mitral valve vegetation. The diagnosis of IE was made and flucloxacillin was initiated. Clinical suspicion was decisive for diagnosis.

This case illustrates a serious and atypical presentation of an already uncommon disease in a patient without known risk factors for IE. While the initial cardiology workup was negative, a high clinical suspicion should always motivate further investigation as the consequences of untreated acute endocarditis are serious and life-threatening.

## Introduction

Infective endocarditis (IE) is a relatively uncommon disease that can manifest as a spectrum of clinical findings. Clinicians should be aware of the wide range of manifestations to reach a quick diagnosis, as delay could be fatal. We present the following case in accordance with the CARE (CAse REports) reporting checklist.

## Case presentation

**Figure 1:**
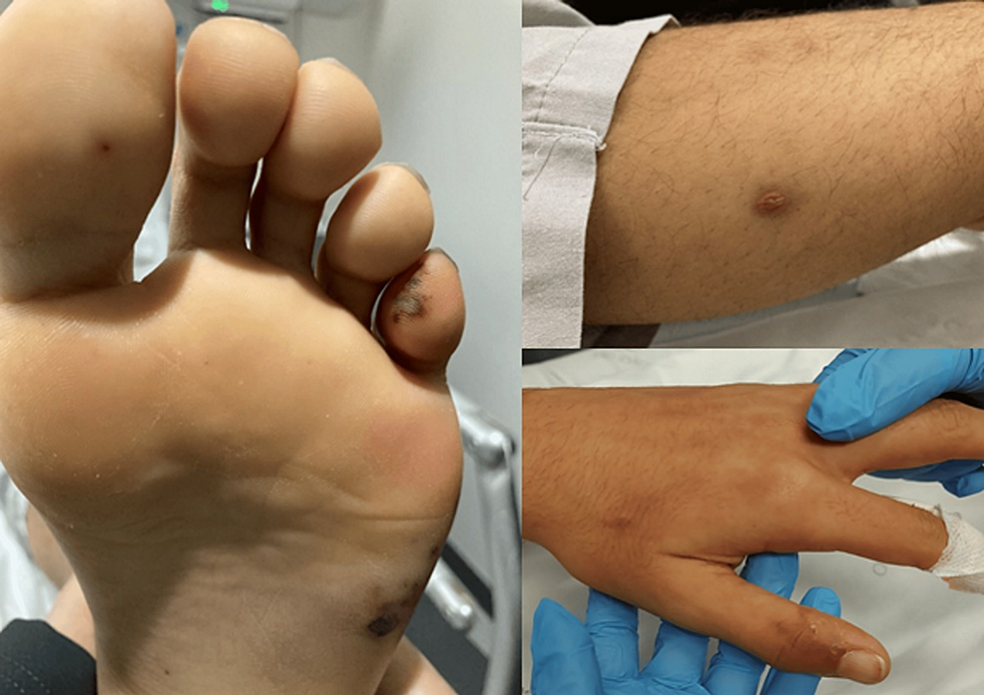
Skin lesions suggestive of Osler’s nodes

A 14-year-old adolescent was admitted to the emergency department due to three days of fever (39.6ºC maximum axillary temperature), coughing, small-joint pain (right radiocarpal joint and bilateral tibiotalar joints), and blurred vision. He had no other complaints. There was no relevant medical, epidemiological, or familial history. Objectively, the patient was febrile, shivering, tachycardic, and normotensive. A hemorrhagic lesion on the sole and edema of the 5th finger were noted on the left foot (Figure 1).

Pulmonary and cardiac auscultation was unremarkable. Neck rigidity and an overall decrease in muscle strength were the only noted changes in the neurological examination at admission.

**Figure 2:**
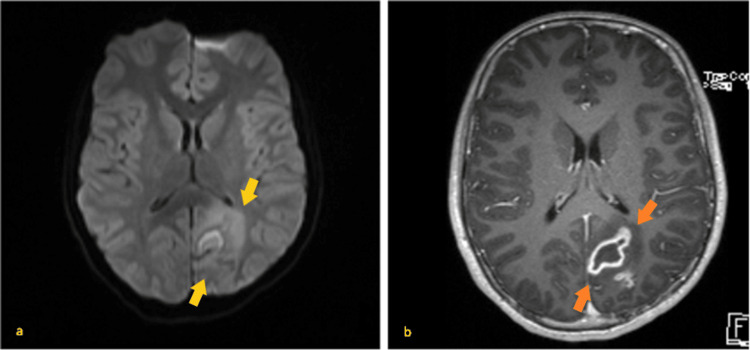
Brain MRI (a) Brain MRI showing a brain mass in the left parieto-occipital paramedian area, with mass effect, vasogenic edema, and restriction to diffusion (yellow arrows). (b) On T1-weighted, contrast-enhanced imaging, peripheral hyperenhancement can be noted (orange arrows).

The patient was started on ceftriaxone (2 g intravenous bolus, every 12 hours) after collecting two sets of blood cultures (one from each arm) and underwent a lumbar puncture (86 leukocytes/μL, 73% neutrophils, 0.49 g/L of proteins, 72 mg/dL of glucose, and negative for herpes simplex virus, enterovirus, and bacteria). Blood work showed leukocytosis (12160/uL), blood glucose of 121 mg/dL, elevated aspartate and alanine aminotransferases (<2 times the upper limit of normal), increased C-reactive protein (201 mg/L), and increased troponin I (179 ng/L; normal range: 0-40 ng/L). The increase in troponin led to an echocardiogram that found no relevant changes. In the emergency department, the foot suffusion progressed to the leg and he developed a right-sided lip drooping. A head CT scan showed cortico-subcortical parietal parasagittal left hypodense lesions, suspected to be ischemic in origin. An additional body CT scan showed pulmonary and kidney ischemic lesions. He was admitted to the pediatric ward, and acyclovir, clindamycin, and gentamicin were added, as primary central nervous system infection was suspected at this point. A brain MRI (Figure 2) described multiple T2-hyperintense lesions in the left occipitoparietal lobes, right temporal lobe, and right frontal lobe. All lesions showed restriction to diffusion while one showed peripheral hyperenhancement after contrast.

**Figure 3:**
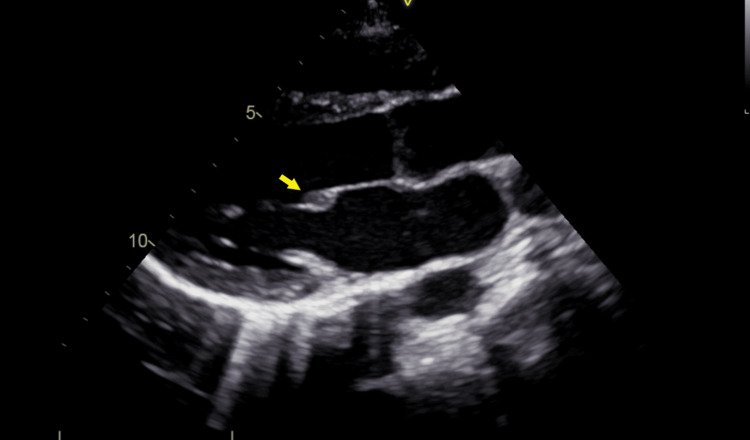
Transthoracic echocardiogram Transthoracic echocardiogram showing a 7 x 7 mm hypoechogenic vegetation on the ventricular side of the anterior leaflet of the mitral valve (yellow arrow).

On the third day of admission, as both sets of aerobic cultures came back positive for methicillin-sensitive *Staphylococcus aureus*, an infectious endocarditis was heavily suspected. As two transthoracic echocardiograms were normal, a transesophageal echocardiogram was performed and showed a 7 x 7 mm hypoechogenic vegetation on the ventricular side of the anterior leaflet of the mitral valve. A subsequent transthoracic echocardiogram was able to identify the described vegetation (Figure 3).

The antibiotic therapy was de-escalated to flucloxacillin 2 g every six hours (total of 8 g/day). On the 9th day of admission, the adolescent developed sudden right-sided inferior quadrantanopia. These acute changes led to a CT scan, which showed that the previously known parieto-occipital lesion had evolved into an abscess (22 x 14 mm) with a local mass effect. As there was no further clinical deterioration and the abscess decreased in size in further evaluations, no direct intervention was performed. The patient completed eight weeks of parenteral flucloxacillin with analytical resolution, MRI improvement, and clinical stability. Nevertheless, inferior quadrantanopia persists at two years of follow-up.

## Discussion

IE, while common in older adults, is a rare disease in pediatric patients. Incidence rates are similar in the developed world, ranging from 0.38 cases/year/100,000 inhabitants in the Portuguese population to 0.43 cases/year/100,000 inhabitants in the United States [1,2]. The incidence rates have been slowly increasing for the last 20 years, owing probably to better clinical outcomes and increasing survival of pediatric patients with complex congenital heart disease [3].

Most patients with IE have associated risk factors such as congenital heart disease (with or without previous corrective surgery) and/or indwelling devices, but 8-10% have no identifiable risk factor [4]. This absence of risk factors for IE, as in our case, constitutes a potential pitfall that usually delays diagnosis and highlights the need for deeper diagnostic investigation in patients with persistent fevers. In such patients, left-sided endocarditis with *S. aureus* bacteremia is the most common presentation [3]. Understanding if the patient has underlying heart disease is vital, as it defines the most common causative pathogens. In patients without heart disease, *S. aureus* is found in almost 50% of cases while *Streptococcus viridans* follows in 18% of cases. In patients with underlying conditions, *S. viridans* is responsible for 33% while *S. aureus* is present in 28% of all cases [1].

The clinical picture of IE in pediatric patients is dependent upon causative microorganisms, the underlying (or absence of) cardiac disease, the degree of embolization, and the presence of immunological manifestations. Thus, disease presentation may be highly variable between patients, requiring a high degree of suspicion for the diagnosis and a multidisciplinary approach. Our case was paradigmatic because of its significant neurological presentation.

IE can be subdivided into acute (high fevers, rapidly progressive, and fulminant disease) or subacute forms (low-fevers and non-specific complaints, usually in patients with underlying cardiac disease). Fever, tachycardia, and hypotension are usually present. Valvulitis usually results in a new or changing heart murmur. Some patients may manifest acute heart failure due to valve perforation, chordal rupture, or decreased ventricular function. Immune-related glomerulonephritis is common in children while other immune-related phenomena such as Roth's spots, Janeway lesions, and Osler nodes are rare in children with IE. Septic embolism is relatively common in children, resulting in extra-cardiac infection (common in the lung or bone, rare in the brain) or infarction (common in the brain, kidney, gastrointestinal tract, and lungs) [5]. We believe our case highlights the common manifestations in children (embolism and fever) while also demonstrating some rarer manifestations such as immune-mediated skin lesions and the absence of risk factors.

When IE is suspected, blood cultures should be obtained as soon as possible so that antimicrobial therapy can be started without delay. The choice of antibiotic, dosing, and duration of treatment are dependent on the microorganism and the presence of risk factors.

As our patient was positive for methicillin-sensitive *S. aureus*, treatment with flucloxacillin was pursued for six weeks, according to the European Society of Cardiology guidelines [6].

Surgical treatment should be considered in all patients with heart failure and progressive disease despite correct antibiotic therapy and embolic phenomena. Data for children are scarce and surgical management is mostly dictated by adult guidelines [5].

In children, mortality associated with *S. aureus* IE ranges from an average of 2.8% to a reported maximum of 4.7% [1]. Complications associated with IE are uncommon in children and are usually associated with risk factors such as prosthetic valves, left-side involvement, *S. aureus* infection, and congenital heart disease. Cardiac dysfunction, metastatic infection, stroke, and acute kidney injury are among the described complications of IE, albeit uncommon in children [1,5].

## Conclusions

This case illustrates a serious and atypical presentation of an already uncommon disease in a patient without known risk factors for IE. While the initial cardiology workup was negative, a high clinical suspicion should always motivate further investigation, as the consequences of untreated acute endocarditis are severe and life-threatening.
